# The origins and drivers of sexual size dimorphism in sharks

**DOI:** 10.1002/ece3.11163

**Published:** 2024-03-17

**Authors:** Joel H. Gayford, Phillip C. Sternes

**Affiliations:** ^1^ Department of Life Sciences Silwood Park Campus, Imperial College London London UK; ^2^ Shark Measurements London UK; ^3^ Department of Evolution, Ecology and Organismal Biology University of California Riverside California USA

**Keywords:** Chondrichthyes, ecology, Elasmobranchii, natural selection, sexual conflict, sexual selection

## Abstract

While sexual size dimorphism (SSD) is abundant in nature, there is huge variation in both the intensity and direction of SSD. SSD results from a combination of sexual selection for large male size, fecundity selection for large female size and ecological selection for either. In most vertebrates, it is variation in the intensity of male–male competition that primarily underlies variation in SSD. In this study, we test four hypotheses regarding the adaptive value of SSD in sharks—considering the potential for each of fecundity, sexual, ecological selection and reproductive mode as the primary driver of variation in SSD between species. We also estimate past macroevolutionary shifts in SSD direction/intensity through shark phylogeny. We were unable to find evidence of significant SSD in early sharks and hypothesise that SSD is a derived state in this clade, that has evolved independently of SSD observed in other vertebrates. Moreover, there is no significant relationship between SSD and fecundity, testes mass or oceanic depth in sharks. However, there is evidence to support previous speculation that reproductive mode is an important determinant of interspecific variation in SSD in sharks. This is significant as in most vertebrates sexual selection is thought to be the primary driver of SSD trends, with evidence for the role of fecundity selection in other clades being inconsistent at best. While the phylogenetic distribution of SSD among sharks is superficially similar to that observed in other vertebrate clades, the relative importance of selective pressures underlying its evolution appears to differ.

## Graphical abstract

Sexual size dimorphism in vertebrates is thought to occur due to sexual selection and/or fecundity selection in most species. In this study, we analyse patterns of SSD in sharks, an ancient clade with unparalleled variation in reproductive biology. We find that early sharks likely did not exhibit SSD and that the selective factors underlying SSD trends in sharks differ from those seen in other vertebrates. SSD in sharks is determined predominantly by reproductive mode.

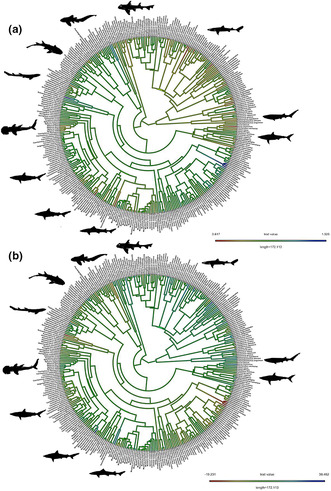

## 1 INTRODUCTION

Differences in body size between organisms have an array of physiological, biomechanical, ecological and evolutionary implications (Blanckenhorn, 2000; Brown et al., 1993; Woodward et al., 2005). Such differences are of course present between taxa but also between the sexes—a phenomenon referred to as sexual size dimorphism or SSD (Horne et al., 2020). SSD is abundant in the natural world and has been studied in a range of taxa (Monnet & Cherry, 2002; Parker, 1992; Shine, 1994). The magnitude and direction of SSD are known to be highly variable, as in some species females reach larger sizes (female‐biased SSD), whereas in others, it is males that are typically larger (Isaac, 2005; Rohner et al., 2018; Webb & Freckleton, 2007). Empirical studies and life‐history theory predict that variability in SSD (both in terms of direction and magnitude) is likely to be driven by the relative strengths of sexual selection, fecundity selection and ecological selection (Fairbairn et al., 2007). Strong fecundity selection is thought to result in female‐biased SSD, whereas strong sexual selection (specifically male–male competition) is thought to favour the evolution of male‐biased SSD (Fairbairn et al., 2007; Head, 1995; Janicke & Fromonteil, 2021; Parker, 1992) and ecological selection can favour either male‐biased or female‐biased SSD (Main et al., 1996; Shine, 1989; Wearmouth & Sims, 2008). In all cases, these are general trends, and examples are known of sexual selection favouring smaller males or variation in reproductive tactics as opposed to male‐biased SSD (Pilastro et al., 1997). In reality, it is likely that many selective pressures contribute to the evolution of SSD, but current studies are primarily restricted to inferences on the basis of correlation between SSD and various facets of life history and ecology. The degree to which each of these processes appears to influence SSD in different clades differs greatly, and only through rigorous empirical studies of SSD and its correlates can we hope to understand its adaptive value (Horne et al., 2020). In light of the controversy regarding selective drivers of SSD in vertebrates and historic bias towards certain clades (Pincheira‐Donoso & Hunt, 2017) there is a need for additional studies of SSD targeting diverse radiations throughout vertebrate phylogeny.

Sharks (Elasmobranchii: Selachii) are a morphologically and ecologically disparate group of vertebrates distributed globally in marine and freshwater ecosystems (Heithaus et al., 2010; Heupel et al., 2014; Sternes & Shimada, 2020). Sharks exhibit a vast degree of variation in life‐history parameters and reproductive biology, making them ideal candidate taxa for the study of SSD and its evolutionary drivers (Carrier et al., 2004; Cortés, 2000). Moreover, as the sister taxon to Osteichthyes, macroevolutionary trends within Chondrichthyes are of great relevance to our understanding of character transitions and trait evolution in jawed vertebrates (Hara et al., 2018; Stein et al., 2018; Venkatesh et al., 2014). Sexual dimorphism is abundant in sharks (Gayford, 2023), and indeed, SSD has been reported in many species (Colonello et al., 2020; Sims, 2005). Existing studies have speculated that the magnitude and direction of SSD in sharks may relate to differences in reproductive mode and/or the intensity of sexual selection (Colonello et al., 2020; Sims, 2005). The first of these hypotheses suggests that selection on large female body size may be relaxed in oviparous taxa relative to matrotrophic taxa due to the external development of embryos and extensive reproductive period (Sims, 2005). It has also been suggested that sexual selection is more intense in oviparous elasmobranchs, and that this would favour the evolution of male‐biased SSD (Colonello et al., 2007, 2020). Indeed, in most vertebrate groups, it has been found that variation in SSD between species is primarily driven by variation in the intensity of male–male competition (Fairbairn et al., 2007; Head, 1995; Horne et al., 2020; Parker, 1992). While sexual conflict and sexual selection are thought to be abundant in elasmobranchs (Gayford, 2023; Rowley et al., 2019), a lack of data have impeded our understanding of macroevolutionary changes in these traits, and the extent to which they might correlate with SSD. Importantly, given the abundance of sexual segregation in elasmobranchs (Mucientes et al., 2009), and the high variation in fecundity between species (Cortés, 2000, 2008), it is likely that all three of these factors contribute to the evolution of SSD, and one study considering ‘fishes’ (including several elasmobranch species) found that the observed distribution of SSD was consistent with selection for increased male size rather than fecundity selection (Horne et al., 2020), however, the predominance of bony fish in this data set means that there is little reason to suggest these results should be indicative of the evolutionary drivers of SSD operating through shark phylogeny.

In this study, we use comparative phylogenetic methods and a large, diverse data set to investigate the evolution of SSD in sharks—an ancient group displaying a broad array of life‐histories and reproductive modes. Primarily, we fit different evolutionary models to test the following four hypotheses: that post‐copulatory sexual selection varies systematically with the strength and/or direction of SSD (1), that fecundity selection varies systematically with the strength and/or direction of SSD (2), that ecology varies systematically with the strength and/or direction of SSD (3) and that reproductive mode varies systematically with the strength and/or direction of SSD (4). We also perform evolutionary model tests and ancestral state reconstruction to provide insight into the evolutionary dynamics underlying macroevolutionary shifts in SSD. Importantly, while we do not directly measure selection in any form, to test hypotheses relating to sexual and fecundity selection, we use life‐history traits generally thought to covary strongly with these selective regimes. This is the first rigorous quantitative study to address the evolution of SSD in sharks, directly addressing speculative hypotheses that have arisen in the literature. Our analyses uncover systematic patterns in the distribution of SSD defined by differences in reproductive mode but fail to recover any evidence of differences in SSD delineated by sexual selection, reproductive output or depth. Given the phylogenetic placement of sharks and the degree of life‐history variation they exhibit, these results are of paramount importance to our understanding of the adaptive value of SSD and sexual dimorphism more broadly.

## 2 METHODOLOGY

### 2.1 Data collection

The following biological data were extracted from the reference book ‘Sharks of the World: a Complete Guide’ (Ebert et al., 2021): maximum length (cm), length at birth (cm), length at maturity for each sex (cm), reproductive mode (matrotrophic or oviparous), minimum litter size, maximum litter size, habitat type (benthic, benthopelagic, pelagic) and depth (shallow, intermediate, deep). For oviparous taxa, eggcase length was also recorded. Quantitative measures of minimum, median and maximum depth were also gathered from FishBase (Froese & Pauly, 2023). For all measurements if upper bounds were unknown, the lower bound was taken as the default value. Where unverified size records existed, they were ignored, and where a range of values were provided for a given measurement the median value was taken. Species for which the length of maturity for both sexes and/or maximum length were not known were excluded, as were species whose phylogenetic placement remains unresolved. The relative strength of sexual selection was modelled as body size‐corrected testes mass (a common proxy for post‐copulatory sperm competition) and data were extracted from Rowley et al. (2019).

Two measures of SSD were defined using the body size data extracted from ‘Sharks of the World: a Complete Guide’ (Ebert et al., 2021): male‐to‐female ratio (MFR) is the ratio of median length at sexual maturity between males and females, and sexual dimorphism percentage (SD%) is the percentage of maximum total length corresponding to the difference in median length at sexual maturity between the two sexes. SD% measures the magnitude of SSD, whereas MFR measures the direction of SSD. Thus, the absence of SSD would result in an MFR value of one and an SD% value of zero. To take into account size variation between taxa, length at birth and eggcase length were standardised, dividing them by maximum total length. Minimum, median and maximum litter sizes were multiplied by this standardised value of neonate/eggcase length (depending on whether the taxon in question was matrotrophic or oviparous) to provide a more reasonable estimate of reproductive output. These measures will henceforth be referred to as minimum, median and maximum reproductive output.

**FIGURE 1:**
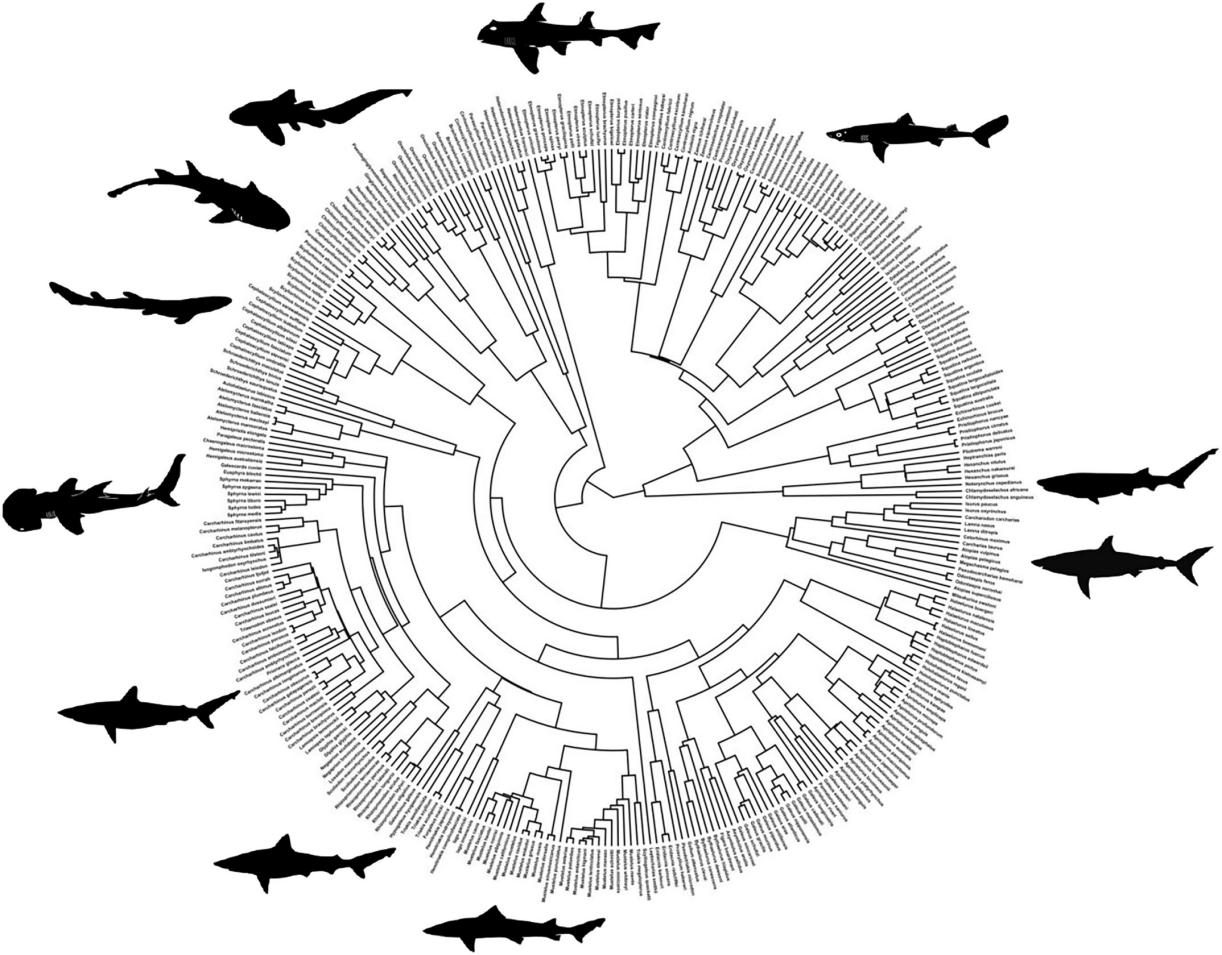
Time‐scaled molecular phylogeny displaying the taxa and interrelationships utilised in this study. Branch lengths were obtained from Stein et al. (2018). Silhouettes are representative taxa from various selachimorph radiations included in the study. Silhouette images obtained online have been dedicated to the public domain.

Phylogenetic data were extracted from Stein et al. (2018), and the resulting time‐scaled phylogeny was pruned to match the data using the function *match.phylo.data* in the *R* package *picante* (Kembel et al., 2010). The final data set includes 339 taxa representing a range of ecologies and including members of all major selachimorph radiations (Figure 1).

### 2.2 Data analysis

All analyses were carried out in the *R* statistical environment (R Core Team, 2023). Prior to analyses, the variables raw minimum litter size, raw median litter size, raw maximum litter size, minimum reproductive output, median reproductive output and maximum reproductive output were log‐transformed. Minimum depth (m), median depth (m) and maximum depth (m) were also log‐transformed, however, due to the presence of zero values, a constant value of 1 was added to all values prior to log‐transformation. Testes mass was not log‐transformed due to low skew and the presence of negative values. Reproductive mode was coded as a binary variable with matrotrophy represented by a value of 1, and oviparity represented by a value of 0.

To compare different adaptive hypotheses for the evolution of SSD, we fit a series of phylogenetic linear models to our data using the package *phylolm* (Ho et al., 2016), testing for possible evolutionary relationships between SSD (SD% and MFR) and potential biological/ecological correlates. For each of SD% and MFR, 8 models were fit initially, each including one of the following covariates: reproductive mode, testes mass, minimum reproductive output, median reproductive output, maximum reproductive output, minimum depth (m), median depth (m), and maximum depth (m). An Ornstein–Uhlenbeck (OU) covariance model was used to provide the phylogenetic correction for these models. OU‐based models of trait variation are typically a prerequisite for testing adaptive hypotheses, as unlike Brownian motion models they allow traits to evolve towards one or more optima (Cressler et al., 2015). In all cases, 100 independent bootstrap replicates were generated.

Due to discrepancies in the number of taxa for which potential biological/ecological correlates are known, we subsequently repeated these analyses using a reduced sample size, permitting direct AIC‐based comparison of the resulting models. We also fit more complex phylogenetic linear models including all possible combinations of the following covariates: reproductive mode, depth (the depth variable with the most explanatory AIC value was selected), reproductive output (the reproductive output variable with the most explanatory AIC value was selected). To provide a comparative baseline by which to assess these models, we also fit null models without any covariates. We chose this approach instead of fitting models with all possible combinations to reduce model redundancy. Testes mass was excluded from these combined models as the small number of taxa for which this parameter is known would substantially reduce the statistical power of these analyses, which require equivalent sample sample size for AIC‐based model selection.

To test the validity of assumptions made by OU‐based models, we used the package mvMORPH (Clavel et al., 2015) to fit single (BM1 and OU1) and multi‐peak (BMM and OUM) BM and OU models to both SSD parameters (SD% and MFR), selecting the best‐supported model of trait evolution on the basis of AICc values (Posada & Buckley, 2004). Finally, to map the evolutionary history of SD% and MFR, ancestral state reconstruction was performed upon both traits in the package *phytools* (Revell et al., 2008).

All data used in this study can be found in the Supporting Information associated with the article.

## 3 RESULTS

Our evolutionary model test revealed that in the cases of both SD% and MFR, Ornstein–Uhlenbeck (OU) models of trait evolution better explain the phylogenetic distribution of SSD than Brownian motion (BM) models (Table 1). Multi‐peak OU models received the most support, whereas single‐peak BM models received the least support (Table 1).

**TABLE 1:** Output from the evolutionary model test, including difference covariance models fit to SSD data and values for model support (AICc, log‐likelihood).

SSD measure	Covariance model	Log‐likelihood	AICc	ΔAICc
SD%	BM1	−621.3745	1247	–
SD%	BMM	−575.2937	1159	−88
SD%	OU1	−458.3395	925	−322
SD%	OUM	−323.1288	659	−588
MFR	BM1	114.1443	−224	–
MFR	BMM	117.0442	−226	−2
MFR	OU1	240..8408	−474	−250
MFR	OUM	351.1598	−690	−466

*Note*: See methodology for details of the covariance models.

Abbreviations: AIC, Akaike information criteria; MFR, male‐to‐female ratio; SSD, sexual size dimorphism.

Univariate phylogenetic linear models using the full data set recovered one statistically significant relationship between SSD potential covariates (Table 3). Reproductive output correlated significantly with both SD% and MFR (Table 3). All other relationships were non‐significant and could not be compared on the basis of AIC due to differential sample sizes.

Univariate phylogenetic linear models using a reduced data set failed to recover any statistically significant relationships between SSD and other potential covariates (Table 3). In the case of both SD% and MFR, null models excluding all covariates received greater support than any covariate models (Table 2).

**TABLE 2:** Output from phylogenetic linear models using the full data set, including the evolutionary parameters *α* and $\sigma^{2}$.

SSD measure	Covariate	Scaling coefficient	*p* value	Log‐likelihood	AICc	*α*	$\sigma^{2}$
SD%	Testes mass	0.322321	.9630803	−58.47	126.95	7.41e−03	6.94e−01
SD%	Minimum depth	0.0509014	.0773	−381.3	772.6	3.47e−03	1.78e−03
SD%	Median depth	0.045666	.4182	−380.3	770.6	3.94e−03	1.75e−03
SD%	Maximum depth	0.0084177	.8738	−409.6	829.1	4.22e−03	2.05e−03
SD%	Minimum reproductive output	0.113094	.1482	−240.7	491.3	2.63e−03	2.10e−03
SD%	Median reproductive output	0.074045	.4954	−238.1	486.2	2.67e−03	1.80e−03
SD%	Maximum reproductive output	−0.0026656	.9776	−247.3	504.5	3.37e−03	2.56e−03
SD%	Reproductive mode	3.60979	.03465	−1081	2171	3.45e−03	0.2011159
MFR	Testes mass	−0.039473	.6305	21.47	−32.93	6.56e−03	8.86e−05
MFR	Minimum depth	−0.0029959	.4124	227.7	−445.4	1.68e−03	3.15e−05
MFR	Median depth	−0.0092905	.1993	228.4	−446.8	1.96e−03	3.02e−05
MFR	Maximum depth	−0.0040548	.5510	241.6	−473.3	2.43e−03	4.04e−05
MFR	Minimum reproductive output	−0.0117264	.21492	160.0	−309.9	1.46e−03	2.40e−05
MFR	Median reproductive output	−0.021151	.1099	158.6	−307.3	1.60e−03	2.36e−05
MFR	Maximum reproductive output	−0.013800	.22238	164 3	−318.6	1.68e−03	2.53e−05
MFR	Reproductive mode	−0.053258	.01923	296.7	−583.3	3.32e−03	4.078e−05

*Note*: AIC values cannot be compared due to non‐equivalent sample sizes between models.

Multivariate phylogenetic linear models did not receive greater support than the null model in either the case of SD% or MFR (Tables 3 and 4). In both cases, multivariate models containing reproductive mode and reproductive output only received more support than those incorporating depth (Table 4). While none of these models were favoured over the null model, a significant relationship between median reproductive output and MFR was found in the model incorporating reproductive output and reproductive mode (Table 4).

**TABLE 3:** Output from phylogenetic linear models using the reduced data set, including the evolutionary parameters *α* and $\sigma^{2}$.

SSD measure	Covariate	Scaling coefficient	*p* value	Log‐likelihood	AICc	*α*	$\sigma^{2}$
SD%	None (null)	NA	NA	−205.9	419.7	2.04e−03	1.46e−03
SD%	Minimum depth	0.040111	.294	−205.3	420.6	2.25e−03	1.39e−03
SD%	Median depth	0.031405	.6567969	−205.8	421.6	2.20e−03	1.38e−03
SD%	Maximum depth	0.018139	.793286	−205.8	421.7	2.13e−03	1.41e−03
SD%	Minimum reproductive output	0.078698	.3395	−205.4	420.8	2.23e−03	1.54e−03
SD%	Median reproductive output	0.069694	.5373	−205.7	421.3	2.19e−03	1.50e−03
SD%	Maximum reproductive output	0.003578	.9717	−205.9	421.7	2.05e−03	1.46e−03
SD%	Reproductive mode	0.858564	.2157	−205.1	420.2	2.23e−03	1.41e−03
MFR	None (null)	NA	NA	134.8	−261.5	2.27e−03	2.05e−05
MFR	Minimum depth	0.00021796	.96328	134.8	−259.5	1.22e−03	2.05e−05
MFR	Median depth	−0.0090363	.2958	135.2	−260.5	1.46e−03	1.84e−05
MFR	Maximum depth	−0.0079795	.345	135.2	−260.3	1.42e−03	1.86e−05
MFR	Minimum reproductive output	−0.008537	.39570	135.1	−260.2	1.27e−03	2.04e−05
MFR	Median reproductive output	−0.023748	.08475	136.3	−262.5	1.37e−03	2.07e−05
MFR	Maximum reproductive output	−0.017577	.1520	135.8	−261.6	1.32e−03	1.99e−05
MFR	Reproductive mode	−0.085913	.3170	135.3	−260.5	1.29e−03	1.98e−05

*Note*: AIC values can be compared directly due to equivalent sample sizes underlying all models.

Abbreviations: AIC, Akaike information criteria; MFR, male‐to‐female ratio; SSD, sexual size dimorphism.

**TABLE 4:** Output from multivariate phylogenetic linear models using the reduced data set, including the evolutionary parameters *α* and $\sigma^{2}$.

SSD measure	Covariates	Scaling coefficients	*p* values	Log‐likelihood	AIC	α	$\sigma^{2}$
SD%	Minimum depth, reproductive mode	0.044437, 0.916676	.2422, .1816	−204.4	420.9	2.52e−03	1.33e−03
SD%	Minimum depth, minimum reproductive output	0.036634, 0.070119	.3414, .3969	−205.0	421.9	2.43e−03	1.47e−03
SD%	Minimum depth, minimum reproductive output, reproductive mode	0.040833, 0.093483, 1.064259	.2837, .2645, .1274	−203.8	421.6	2.85e−03	1.41e−03
SD%	Minimum reproductive output, reproductive mode	0.101246, 1.022244	.2257, .1476	−204.4	420.7	2.54e−03	1.50e−03
MFR	Minimum depth, reproductive mode	−0.010104, −0.093573	.2413, .2663	135.9	−259.7	1.60e−03	1.74e−05
MFR	Minimum depth, median reproductive output	−0.0067935, −0.0220288	.4357, .1129	136.5	−261.1	1.57e−03	1.90e−05
MFR	Minimum depth, median reproductive output, reproductive mode	−0.0079247, −0.0268892, −0.1318313	.35852, .05779, .12472	137.7	−261.4	1.81e−03	1.75e−05
MFR	Median reproductive output, reproductive mode	−0.028617, −0.128467	.04253, .14125	137.3	−262.7	1.52e−03	1.95e−05

*Note*: AIC values can be compared directly due to equivalent sample sizes underlying all models.

Abbreviations: AIC, Akaike information criteria; MFR, male‐to‐female ratio; SSD, sexual size dimorphism.

**FIGURE 2:**
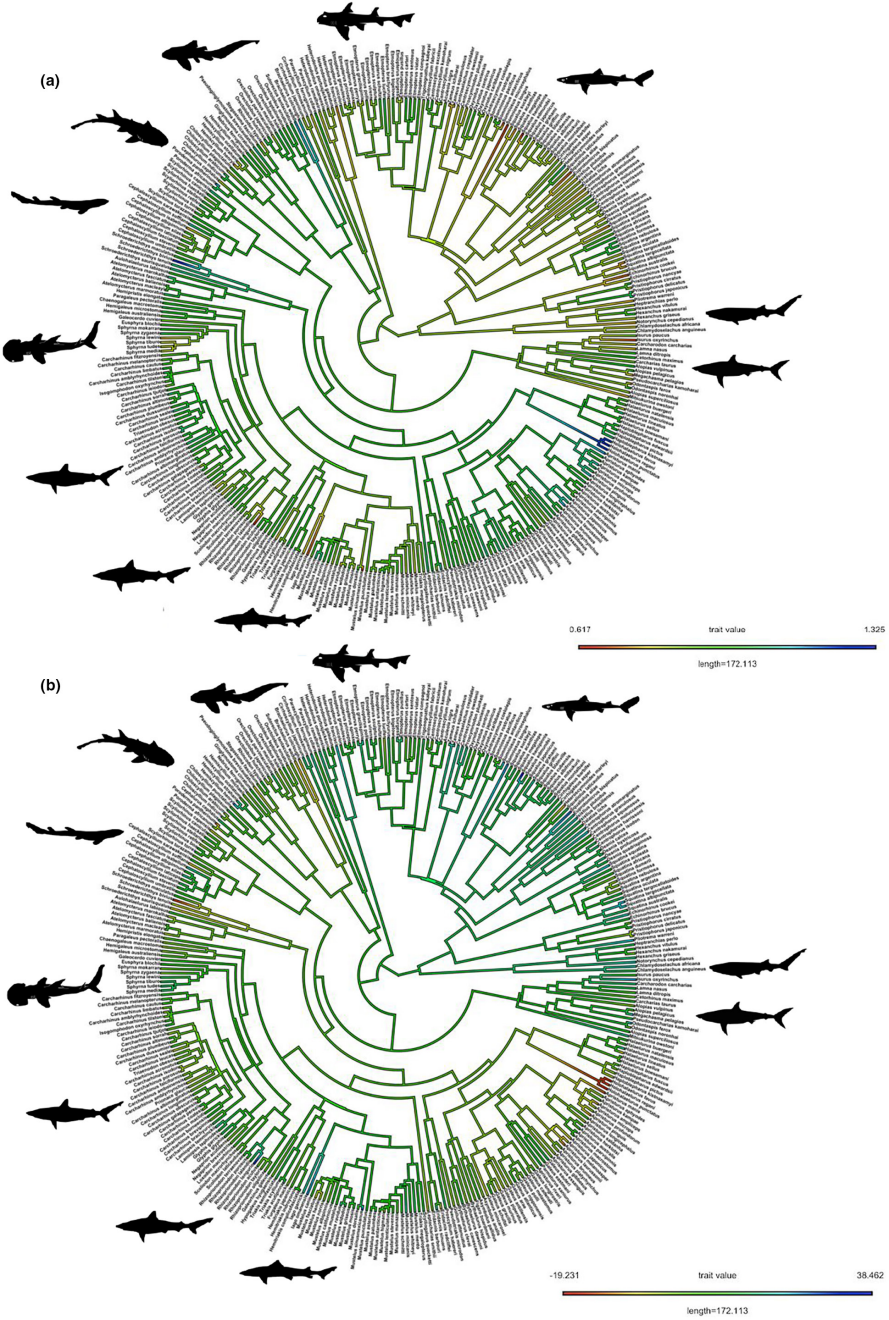
Ancestral state reconstructions for sexual dimorphism as male–female ratio (a) and a percentage of maximum total length (b) superimposed upon the selachimorph phylogeny, displaying the evolutionary histories of these two traits.

Ancestral state reconstruction of both SSD measures estimated an ancestral selachian with an SD% value of 10.80 (95% CI: 0≤x≥34.05) and an MFR value of 0.868 (95% CI: 0.59≤x≥1.15), with multiple independent increases and decreases in sexual dimorphism occurring since (Figure 2). There is thus no evidence to suggest that the male and female ancestral selachimorphs differed significantly in size.

## 4 DISCUSSION

In this study, we aimed to improve our understanding of the evolution of SSD in vertebrates by testing for relationships between the direction and magnitude of SSD and (1) the intensity of sexual selection, (2) the intensity of fecundity selection, (3) ecology and (4) reproductive mode in sharks. We also estimated past macroevolutionary shifts in SSD magnitude/direction across shark phylogeny. Contrary to other vertebrate clades, we fail to recover any evidence for relationships between SSD and fecundity, sexual selection or ecology (Tables 2, 3, 4). However, we do find evidence to support previous speculation that there exists some relationship between SSD and reproductive mode (Colonello et al., 2020). Regarding the evolutionary origins of SSD in sharks, we rather surprisingly fail to recover evidence of significant SSD in early selachians. In the following sections, we expand on these results, what they might tell us about the evolution of SSD in vertebrates, and what future advances will be necessary to better understand trends in SSD among cartilaginous fishes.

### 4.1 SSD and reproductive mode: Morphological constraint or sexual selection?

Our results support a previous, speculative hypothesis that reproductive mode is an important determinant of interspecific variation in SSD among shark species (Table 2). Indeed, our results suggest that oviparous taxa are associated with relatively low SD% values and relatively large MFR values, whereas matrotrophic taxa (including placental and aplacental viviparity) have relatively large SD% values and relatively low MFR values (Table 2). This suggests that male‐biased SSD is more prevalent in oviparous shark species, whereas viviparous taxa are more likely to exhibit female‐biased SSD. It is also worth noting that SSD is generally less intense in oviparous species compared to viviparous species, as evidenced by differential SD% values (Table 2). While these significant results were not recovered in subsequent analyses (Tables 3 and 4), this is not surprising as only one oviparous taxon was included in the reduced data set. The role of reproductive mode in determining the direction of SSD in sharks has been mentioned in previous studies (Colonello et al., 2020; Sims, 2005), however, until now this has been a purely speculative hypothesis, with no phylogenetically‐informed evidence.

Male‐biased SSD was previously suggested to be more prevalent in oviparous taxa for two reasons: relaxed selection on large female body size due to extended reproductive period and external development of embryos (Sims, 2005) and elevated levels of sexual selection for increased male body size relative to matrotrophic species (Colonello et al., 2007, 2020). The first of these factors appears superficially similar to fecundity selection but differs in that it is the spatio‐temporal distribution of reproductive output rather than the magnitude of reproductive effort that differs between species. Selection for increased female size in matrotrophic shark species is logical given the number and size of pups produced and the extended gestation period of many sharks (Au et al., 2008; Tokunaga et al., 2022). Differences in the nature of sexual selection between oviparous and matrotrophic shark species are more problematic: in this study, we focused on testes mass—commonly used as a proxy for the intensity of post‐copulatory sexual selection, and failed to find any relationship between this variable and SSD (Tables 2, 3, 4). Moreover, sexual selection is only thought to favour the evolution of male‐biased SSD, where a high degree of territoriality, sperm competition or parental care is observed (Horne et al., 2020). Direct parental care is unknown in elasmobranchs (Carrier et al., 2004), and indeed would be unexpected given the prevalence of multiple paternity in the clade (Armada‐Tapia et al., 2023). There is no evidence of widespread territoriality in elasmobranchs and although sperm competition is known, this data come from a tiny fraction of extant species (Rowley et al., 2019). Therefore, we suggest that this relationship between reproductive mode and SSD results from relaxed selection of female body size in oviparous taxa (due to spatio‐temporal differences in the distribution of reproductive effort) rather than sexual selection. Importantly, this only provides an explanation for the presence or absence of female‐biased SSD and does not explain cases of male‐biased SSD.

### 4.2 SSD, fecundity and ecology

Despite their importance as a determinant of SSD in other vertebrates, we found no evidence for correlation between the strength/direction of SSD and either fecundity or ecological selection in sharks (Tables 2, 3, 4). Increased female body size is generally thought to convey greater fecundity and reproductive energy output, and thus in species where fecundity selection is stronger, SSD is expected to be greater in magnitude and female‐biased (Head, 1995; Horne et al., 2020; Reeve & Fairbairn, 1999). It is important to note that across vertebrate diversity this hypothesised relationship between fecundity selection and SSD has received inconsistent support, and other factors such as ecological selection are now known to underlie many cases of female‐biased SSD (Pincheira‐Donoso & Hunt, 2017). Our results provide some nuance to ideas of fecundity and reproductive biology influencing SSD, as in a clade with unparalleled variation in reproductive biology, it is spatio‐temporal variation in reproductive output as opposed to reproductive output itself that appears to influence female body size (Table 2). Fecundity data are lacking for the majority of oviparous species however, and additional studies will be required to verify the extent to which fecundity selection may the strength of correlation between different measures of reproductive output and fecundity selection.

Neglected in several evolutionary studies of SSD in fishes (Horne et al., 2020; Parker, 1992), we tested for relationships between an important facet of ecology and both the strength and direction of SSD in sharks but failed to find any significant correlation (Tables 2, 3, 4). It is generally accepted that ecological selection acts on body size in vertebrates (Blanckenhorn, 2000; Sheridan & Bickford, 2011; Shine, 1989), and that in some systems, SSD reflects a balance or trade‐off between natural selection and sexual selection (Nudds & Kaminski, 1984; Pearson et al., 2002; Shine, 1989; Wikelski & Trillmich, 1997). We used depth as a measure of ecology as the shallow‐deep continuum is known to have been an influential force shaping morphological evolution in elasmobranchs (López‐Romero et al., 2023; Sorenson et al., 2014), and it is generally recognised that the complexity and diversity of shallow‐water marine environments (Martinez et al., 2021; Miller et al., 2022) leads to a gradient of relatively weak to strong ecological selection with increasing depth. In shallow‐water environments, greater ecological complexity and higher competition levels could favour enhanced niche/resource partitioning (Cloyed & Eason, 2017), in turn favouring the evolution of stronger SSD, which could be either female or male‐biased depending on the system in question. Resource partitioning has been reported in a number of shark species (Curnick et al., 2019; Kinney et al., 2011) and is known to contribute to SSD in other taxa (Nudds & Kaminski, 1984), but our results suggest that depth is not an important driver of SSD in sharks (Tables 2, 3, 4). It is also worth mentioning that depth is an important determinant of light penetration, which in some fishes shapes female preference and consequently pre‐copulatory sexual selection (Gray et al., 2008; Heinen‐Kay et al., 2015). The role of female preference in sexual selection in sharks is unknown but it is important to recognise that ecological variables such as depth correlate not only with ecological selection but with other potential selective pressures including some facets of sexual selection and reproductive mode (Katona et al., 2023). Additional studies incorporating other facets of ecology are needed, but on the basis of our results, there is no evidence for significant relationships between ecological selection and SSD in sharks.

### 4.3 Macroevolutionary shifts in SSD among sharks and other vertebrates

While the distribution of SSD through shark phylogeny (Figure 2) does not differ fundamentally from that observed in other vertebrate clades, our results have important implications for our understanding of the adaptive value of SSD. The drivers of SSD evolution have been studied in a number of vertebrate groups (including mammals, birds, reptiles, amphibians and fishes) and in most cases, it is sexual selection for increased male size that appears to be the most important factor, with evidence for fecundity selection driven SSD evolution inconsistent or absent despite the dominance of female‐biased SSD (Horne et al., 2020; Monroe et al., 2015; Seehausen et al., 2008). In the absence of vertebrate‐wide comparative phylogenetic studies, this would suggest that broadly across vertebrate diversity sexual selection on male body size is stronger than fecundity selection on female body size. In sharks, it appears that neither of these factors plays an important role in shaping SSD trends (Tables 2, 3, 4), suggesting that the adaptive landscape underlying SSD has undergone a major shift at some point during gnathostome phylogeny.

Intriguingly, our ancestral state reconstruction provided no robust support for SSD in early sharks, with the ancestral values of SD% and MFR overlapping with 0 and 1 respectively. This raises the prospect that SSD in extant selachimorphs is a derived state that has evolved independently from SSD in other vertebrate lineages. This remains to be empirically tested as palaeontological studies of selachimorph taxa rarely comment on sexual size dimorphism. However, given our finding that extant oviparous sharks exhibit less SSD than matrotrophic species (Table 2) and the fact that oviparity likely represents the ancestral reproductive mode in elasmobranchs (Katona et al., 2023), we hypothesise that any SSD found in early selachimorphs would likely be relatively minor if present at all. Subsequently, as alternative, matrotrophic reproductive modes evolved in elasmobranchs, selection on increased female body size was intensified, resulting in the evolution of female‐biased SSD in some lineages. We found that an OUM model of trait evolution best explained the phylogenetic distribution of SSD in sharks (Table 1), suggesting the presence of multiple adaptive peaks which may correspond to male‐biased and female‐biased SSD. The adaptive nature of both male and female‐biased SSD is further evidenced by the fact that both have evolved multiple times independently in extant species (Figure 2). While it is evident that the evolution of female‐biased SSD in sharks was associated with the rise of matrotrophic reproductive modes (Figure 2; Katona et al., 2023), the lack of any relationship between SSD and either ecology or sexual selection means we are unable to speculate as to what selective forces have favoured the repeated evolution of male‐biased SSD. We must emphasise that the evolutionary analyses we have utilised here have a number of underlying assumptions and limitations, relying on phylogenetic hypotheses and specific models of trait evolution. Further studies incorporating additional proxies for sexual selection (both pre‐ and post‐copulatory) and resource partitioning will be necessary to categorically rule out hypothesised drivers of male‐biased SSD in sharks.

## 5 CONCLUSIONS

SSD is undoubtedly abundant in nature, but there remains much controversy over its adaptive value and the selective factors influencing its evolution. Overall, our analyses show that there is no uniform relationship between selection and SSD across vertebrate phylogeny, with superficially similar patterns of SSD in different lineages evolving due to different combinations of selective pressures. It appears that SSD is not ancestral to sharks and may have arisen initially due to the evolution of matrotrophic reproductive modes. Certainly, there is a trend in extant species, whereby oviparous taxa exhibit less, and more male‐biased SSD than matrotrophic taxa, with all available evidence suggesting that this is due to selection on female body size induced by matrotrophy itself, rather than sexual selection. The drivers of male‐biased SSD remain unknown and warrant further study. Sharks represent an important component of vertebrate diversity and occupy a key phylogenetic position within jawed vertebrates, and thus these results are of great importance to our understanding of the adaptive basis of SSD, and how/why broad shifts in SSD trends and underlying drivers may have occurred over vertebrate evolutionary history.

## AUTHOR CONTRIBUTIONS

**Joel H. Gayford:** Conceptualization (lead); data curation (lead); formal analysis (lead); writing – original draft (equal); writing – review and editing (equal). **Phillip C. Sternes:** Conceptualization (supporting); data curation (supporting); writing – original draft (equal); writing – review and editing (equal).

## FUNDING INFORMATION

The authors declare no funding for this study.

## CONFLICT OF INTEREST STATEMENT

The authors declare no conflicts of interest regarding this study.

## Supporting information


Data S1.

## Data Availability

All data generated used during this study can be found within the article and associated Supporting Information.

## References

[ece311163-bib-0068] Armada‐Tapia, S. , Castillo‐Geniz, J. L. , Victoria‐Cota, N. , Arce‐Valdés, L. R. , & Enríquez‐Paredes, L. M. (2023). First evidence of multiple paternity in the blue shark (Prionace glauca). Journal of Fish Biology, 102(2), 528–531. 36401786 10.1111/jfb.15272

[ece311163-bib-0001] Au, D. W. , Smith, S. E. , & Show, C. (2008). Shark productivity and reproductive protection, and a comparison with teleosts. In M. D. Camhi , E. K. Pikitch , & E. A. Babcock (Eds.), Sharks of the open ocean: Biology, fisheries and conservation (pp. 298–308). Wiley‐Blackwell.

[ece311163-bib-0002] Blanckenhorn, W. U. (2000). The evolution of body size: What keeps organisms small? The Quarterly Review of Biology, 75(4), 385–407. 11125698 10.1086/393620

[ece311163-bib-0003] Brown, J. H. , Marquet, P. A. , & Taper, M. L. (1993). Evolution of body size: Consequences of an energetic definition of fitness. The American Naturalist, 142(4), 573–584. 10.1086/285558 19425961

[ece311163-bib-0004] Carrier, J. C. , Pratt, H. L. , & Castro, J. I. (2004). Reproductive biology of elasmobranchs. Biology of Sharks and their Relatives, 10, 269–286.

[ece311163-bib-0005] Clavel, J. , Escarguel, G. , & Merceron, G. (2015). mvMORPH: An R package for fitting multivariate evolutionary models to morphometric data. Methods in Ecology and Evolution, 6(11), 1311–1319.

[ece311163-bib-0006] Cloyed, C. S. , & Eason, P. K. (2017). Niche partitioning and the role of intraspecific niche variation in structuring a guild of generalist anurans. Royal Society Open Science, 4(3), 170060. 28405403 10.1098/rsos.170060 PMC5383860

[ece311163-bib-0007] Colonello, J. H. , Cortés, F. , & Belleggia, M. (2020). Male‐biased sexual size dimorphism in sharks: The narrowmouth catshark Schroederichthys bivius as case study. Hydrobiologia, 847, 1873–1886.

[ece311163-bib-0008] Colonello, J. H. , Lucifora, L. O. , & Massa, A. M. (2007). Reproduction of the angular angel shark (*Squatina guggenheim*): Geographic differences, reproductive cycle, and sexual dimorphism. ICES Journal of Marine Science, 64(1), 131–140.

[ece311163-bib-0009] Cortés, E. (2000). Life history patterns and correlations in sharks. Reviews in Fisheries Science, 8(4), 299–344.

[ece311163-bib-0010] Cortés, E. (2008). Comparative life history and demography of pelagic sharks. Sharks of the Open Ocean: Biology, Fisheries and Conservation, 2, 309–322.

[ece311163-bib-0011] Cressler, C. E. , Butler, M. A. , & King, A. A. (2015). Detecting adaptive evolution in phylogenetic comparative analysis using the Ornstein–Uhlenbeck model. Systematic Biology, 64(6), 953–968. 26115662 10.1093/sysbio/syv043

[ece311163-bib-0012] Curnick, D. J. , Carlisle, A. B. , Gollock, M. J. , Schallert, R. J. , & Hussey, N. E. (2019). Evidence for dynamic resource partitioning between two sympatric reef shark species within the British Indian Ocean Territory. Journal of Fish Biology, 94(4), 680–685. 30784087 10.1111/jfb.13938 PMC6849741

[ece311163-bib-0013] Ebert, D. A. , Fowler, S. , & Compagno, L. (2021). Sharks of the world: A complete guide. Wild Nature Press.

[ece311163-bib-0015] Fairbairn, D. J. , Blanckenhorn, W. U. , & Székely, T. (Eds.). (2007). Sex, size and gender roles: Evolutionary studies of sexual size dimorphism. Oxford University Press.

[ece311163-bib-0016] Froese, R. , & Pauly, D. (2023). FishBase. World Wide Web Electronic Publication. www.fishbase.org

[ece311163-bib-0017] Gayford, J. H. (2023). The evolution of sexual dimorphism in Chondrichthyes: Drivers, uncertainties, and future directions. Environmental Biology of Fishes, 20, 1–13.

[ece311163-bib-0018] Gray, S. M. , Dill, L. M. , Tantu, F. Y. , Loew, E. R. , Herder, F. , & McKinnon, J. S. (2008). Environment‐contingent sexual selection in a colour polymorphic fish. Proceedings of the Royal Society B: Biological Sciences, 275(1644), 1785–1791. 10.1098/rspb.2008.0283 PMC2587793 18445554

[ece311163-bib-0019] Hara, Y. , Yamaguchi, K. , Onimaru, K. , Kadota, M. , Koyanagi, M. , Keeley, S. D. , Tatsumi, K. , Tanaka, K. , Motone, F. , Kageyama, Y. , & Nozu, R. (2018). Shark genomes provide insights into elasmobranch evolution and the origin of vertebrates. Nature Ecology & Evolution, 2(11), 1761–1771. 30297745 10.1038/s41559-018-0673-5

[ece311163-bib-0020] Head, G. (1995). Selection on fecundity and variation in the degree of sexual size dimorphism among spider species (class Araneae). Evolution, 49, 776–781. 28565139 10.1111/j.1558-5646.1995.tb02313.x

[ece311163-bib-0021] Heinen‐Kay, J. L. , Morris, K. E. , Ryan, N. A. , Byerley, S. L. , Venezia, R. E. , Peterson, M. N. , & Langerhans, R. B. (2015). A trade‐off between natural and sexual selection underlies diversification of a sexual signal. Behavioral Ecology, 26(2), 533–542.

[ece311163-bib-0022] Heithaus, M. R. , Frid, A. , Vaudo, J. J. , Worm, B. , & Wirsing, A. J. (2010). Unraveling the ecological importance of elasmobranchs. In Sharks and their relatives II (pp. 627–654). CRC Press.

[ece311163-bib-0023] Heupel, M. R. , Knip, D. M. , Simpfendorfer, C. A. , & Dulvy, N. K. (2014). Sizing up the ecological role of sharks as predators. Marine Ecology Progress Series, 495, 291–298.

[ece311163-bib-0024] Ho, L. S. T. , Ane, C. , Lachlan, R. , Tarpinian, K. , Feldman, R. , Yu, Q. , van der Bijl, W. , Maspons, J. , Vos, R. , & Ho, M. L. S. T. (2016). Package ‘phylolm’. http://cran.r‐project.org/web/packages/phylolm/index

[ece311163-bib-0025] Horne, C. R. , Hirst, A. G. , & Atkinson, D. (2020). Selection for increased male size predicts variation in sexual size dimorphism among fish species. Proceedings of the Royal Society B, 287(1918), 20192640. 31937230 10.1098/rspb.2019.2640 PMC7003453

[ece311163-bib-0026] Isaac, J. L. (2005). Potential causes and life‐history consequences of sexual size dimorphism in mammals. Mammal Review, 35(1), 101–115.

[ece311163-bib-0027] Janicke, T. , & Fromonteil, S. (2021). Sexual selection and sexual size dimorphism in animals. Biology Letters, 17(9), 20210251. 34520680 10.1098/rsbl.2021.0251 PMC8440037

[ece311163-bib-0028] Katona, G. , Szabó, F. , Végvári, Z. , Székely, T., Jr. , Liker, A. , Freckleton, R. P. , Vági, B. , & Székely, T. (2023). Evolution of reproductive modes in sharks and rays. Journal of Evolutionary Biology, 36(11), 1630–1640. 37885147 10.1111/jeb.14231

[ece311163-bib-0029] Kembel, S. W. , Cowan, P. D. , Helmus, M. R. , Cornwell, W. K. , Morlon, H. , Ackerly, D. D. , Blomberg, S. P. , & Webb, C. O. (2010). Picante: R tools for integrating phylogenies and ecology. Bioinformatics, 26(11), 1463–1464. 20395285 10.1093/bioinformatics/btq166

[ece311163-bib-0030] Kinney, M. J. , Hussey, N. E. , Fisk, A. T. , Tobin, A. J. , & Simpfendorfer, C. A. (2011). Communal or competitive? Stable isotope analysis provides evidence of resource partitioning within a communal shark nursery. Marine Ecology Progress Series, 439, 263–276.

[ece311163-bib-0032] López‐Romero, F. A. , Stumpf, S. , Kamminga, P. , Böhmer, C. , Pradel, A. , Brazeau, M. D. , & Kriwet, J. (2023). Shark mandible evolution reveals patterns of trophic and habitat‐mediated diversification. Communications Biology, 6(1), 496. 37156994 10.1038/s42003-023-04882-3 PMC10167336

[ece311163-bib-0033] Main, M. B. , Weckerly, F. W. , & Bleich, V. C. (1996). Sexual segregation in ungulates: New directions for research. Journal of Mammalogy, 77(2), 449–461.

[ece311163-bib-0034] Martinez, C. M. , Friedman, S. T. , Corn, K. A. , Larouche, O. , Price, S. A. , & Wainwright, P. C. (2021). The deep sea is a hot spot of fish body shape evolution. Ecology Letters, 24(9), 1788–1799. 34058793 10.1111/ele.13785

[ece311163-bib-0035] Miller, E. C. , Martinez, C. M. , Friedman, S. T. , Wainwright, P. C. , Price, S. A. , & Tornabene, L. (2022). Alternating regimes of shallow and deep‐sea diversification explain a species‐richness paradox in marine fishes. Proceedings of the National Academy of Sciences, 119(43), e2123544119. 10.1073/pnas.2123544119 PMC9618140 36252009

[ece311163-bib-0036] Monnet, J. M. , & Cherry, M. I. (2002). Sexual size dimorphism in anurans. Proceedings of the Royal Society of London. Series B: Biological Sciences, 269(1507), 2301–2307. 10.1098/rspb.2002.2170 PMC1691160 12495496

[ece311163-bib-0037] Monroe, M. J. , South, S. H. , & Alonzo, S. H. (2015). The evolution of fecundity is associated with female body size but not female‐biased sexual size dimorphism among frogs. Journal of Evolutionary Biology, 28(10), 1793–1803. 26189727 10.1111/jeb.12695

[ece311163-bib-0038] Mucientes, G. R. , Queiroz, N. , Sousa, L. L. , Tarroso, P. , & Sims, D. W. (2009). Sexual segregation of pelagic sharks and the potential threat from fisheries. Biology Letters, 5(2), 156–159. 19324655 10.1098/rsbl.2008.0761 PMC2665836

[ece311163-bib-0040] Nudds, T. D. , & Kaminski, R. M. (1984). Sexual size dimorphism in relation to resource partitioning in north American dabbling ducks. Canadian Journal of Zoology, 62(10), 2009–2012.

[ece311163-bib-0042] Parker, G. A. (1992). The evolution of sexual size dimorphism in fish. Journal of Fish Biology, 41, 1–20.

[ece311163-bib-0043] Pearson, D. , Shine, R. , & How, R. (2002). Sex‐specific niche partitioning and sexual size dimorphism in Australian pythons (*Morelia spilota imbricata*). Biological Journal of the Linnean Society, 77(1), 113–125.

[ece311163-bib-0045] Pilastro, A. , Giacomello, E. , & Bisazza, A. (1997). Sexual selection for small size in male mosquitofish (*Gambusia holbrooki*). Proceedings of the Royal Society of London. Series B: Biological Sciences, 264(1385), 1125–1129.

[ece311163-bib-0046] Pincheira‐Donoso, D. , & Hunt, J. (2017). Fecundity selection theory: Concepts and evidence. Biological Reviews, 92(1), 341–356. 26526765 10.1111/brv.12232

[ece311163-bib-0048] Posada, D. , & Buckley, T. R. (2004). Model selection and model averaging in phylogenetics: Advantages of Akaike information criterion and Bayesian approaches over likelihood ratio tests. Systematic Biology, 53(5), 793–808. 15545256 10.1080/10635150490522304

[ece311163-bib-0049] R Core Team . (2023). R: A language and environment for statistical computing. R Foundation For Statistical Computing.

[ece311163-bib-0050] Reeve, J. P. , & Fairbairn, D. J. (1999). Change in sexual size dimorphism as a correlated response to selection on fecundity. Heredity, 83(6), 697–706. 10651914 10.1046/j.1365-2540.1999.00616.x

[ece311163-bib-0051] Revell, L. J. , Harmon, L. J. , & Collar, D. C. (2008). Phylogenetic signal, evolutionary process, and rate. Systematic Biology, 57(4), 591–601. 18709597 10.1080/10635150802302427

[ece311163-bib-0067] Rohner, P. T. , Teder, T. , Esperk, T. , Lüpold, S. , & Blanckenhorn, W. U. (2018). The evolution of male‐biased sexual size dimorphism is associated with increased body size plasticity in males. Functional Ecology, 32(2), 581–591.

[ece311163-bib-0052] Rowley, A. , Locatello, L. , Kahrl, A. , Rego, M. , Boussard, A. , Garza‐Gisholt, E. , Kempster, R. M. , Collin, S. P. , Giacomello, E. , Follesa, M. C. , & Porcu, C. (2019). Sexual selection and the evolution of sperm morphology in sharks. Journal of Evolutionary Biology, 32(10), 1027–1035. 31250483 10.1111/jeb.13501

[ece311163-bib-0053] Seehausen, O. , Terai, Y. , Magalhaes, I. S. , Carleton, K. L. , Mrosso, H. D. , Miyagi, R. , Van Der Sluijs, I. , Schneider, M. V. , Maan, M. E. , Tachida, H. , & Imai, H. (2008). Speciation through sensory drive in cichlid fish. Nature, 455(7213), 620–626. 18833272 10.1038/nature07285

[ece311163-bib-0054] Sheridan, J. A. , & Bickford, D. (2011). Shrinking body size as an ecological response to climate change. Nature Climate Change, 1(8), 401–406.

[ece311163-bib-0055] Shine, R. (1989). Ecological causes for the evolution of sexual dimorphism: A review of the evidence. The Quarterly Review of Biology, 64(4), 419–461. 2697022 10.1086/416458

[ece311163-bib-0056] Shine, R. (1994). Sexual size dimorphism in snakes revisited. Copeia, 1994, 326–346.

[ece311163-bib-0057] Sims, D. W. (2005). Differences in habitat selection and reproductive strategies of male and female sharks. Sexual Segregation in Vertebrates, 20, 127–147.

[ece311163-bib-0058] Sorenson, L. , Santini, F. , & Alfaro, M. E. (2014). The effect of habitat on modern shark diversification. Journal of Evolutionary Biology, 27(8), 1536–1548. 24890604 10.1111/jeb.12405

[ece311163-bib-0059] Stein, R. W. , Mull, C. G. , Kuhn, T. S. , Aschliman, N. C. , Davidson, L. N. , Joy, J. B. , Smith, G. J. , Dulvy, N. K. , & Mooers, A. O. (2018). Global priorities for conserving the evolutionary history of sharks, rays and chimaeras. Nature Ecology & Evolution, 2(2), 288–298. 29348644 10.1038/s41559-017-0448-4

[ece311163-bib-0060] Sternes, P. C. , & Shimada, K. (2020). Body forms in sharks (Chondrichthyes: Elasmobranchii) and their functional, ecological, and evolutionary implications. Zoology, 140, 125799. 32413674 10.1016/j.zool.2020.125799

[ece311163-bib-0061] Tokunaga, S. , Watanabe, Y. Y. , Kawano, M. , & Kawabata, Y. (2022). Factors affecting gestation periods in elasmobranch fishes. Biology Open, 11(6), bio059270. 35686686 10.1242/bio.059270 PMC9194679

[ece311163-bib-0062] Venkatesh, B. , Lee, A. P. , Ravi, V. , Maurya, A. K. , Lian, M. M. , Swann, J. B. , Ohta, Y. , Flajnik, M. F. , Sutoh, Y. , Kasahara, M. , & Hoon, S. (2014). Elephant shark genome provides unique insights into gnathostome evolution. Nature, 505(7482), 174–179. 24402279 10.1038/nature12826 PMC3964593

[ece311163-bib-0063] Wearmouth, V. J. , & Sims, D. W. (2008). Sexual segregation in marine fish, reptiles, birds and mammals: Behaviour patterns, mechanisms and conservation implications. Advances in Marine Biology, 54, 107–170. 18929064 10.1016/S0065-2881(08)00002-3

[ece311163-bib-0064] Webb, T. J. , & Freckleton, R. P. (2007). Only half right: Species with female‐biased sexual size dimorphism consistently break Rensch's rule. PLoS One, 2(9), e897. 17878932 10.1371/journal.pone.0000897 PMC1964802

[ece311163-bib-0065] Wikelski, M. , & Trillmich, F. (1997). Body size and sexual size dimorphism in marine iguanas fluctuate as a result of opposing natural and sexual selection: An Island comparison. Evolution, 51(3), 922–936. 28568579 10.1111/j.1558-5646.1997.tb03673.x

[ece311163-bib-0066] Woodward, G. , Ebenman, B. , Emmerson, M. , Montoya, J. M. , Olesen, J. M. , Valido, A. , & Warren, P. H. (2005). Body size in ecological networks. Trends in Ecology & Evolution, 20(7), 402–409. 16701403 10.1016/j.tree.2005.04.005

